# Polycrystalline Transparent Al-Doped ZnO Thin Films for Photosensitivity and Optoelectronic Applications

**DOI:** 10.3390/nano13162348

**Published:** 2023-08-16

**Authors:** Victor V. Petrov, Irina O. Ignatieva, Maria G. Volkova, Irina A. Gulyaeva, Ilya V. Pankov, Ekaterina M. Bayan

**Affiliations:** 1 Institute of Nanotechnologies, Electronics, and Equipment Engineering, Southern Federal University, Taganrog 347922, Russia; mvol@sfedu.ru (M.G.V.); iten@sfedu.ru (I.A.G.); 2 Faculty of Chemistry, Southern Federal University, Rostov-on-Don 344090, Russia; iignateva@sfedu.ru (I.O.I.); ekbayan@sfedu.ru (E.M.B.); 3 Institute of Physical and Organic Chemistry, Southern Federal University, Rostov-on-Don 344090, Russia; ipankov@sfedu.ru

**Keywords:** thin film, ZnO, pyrolysis, optical properties, band gap, photosensitive sensors

## Abstract

Thin nanocrystalline transparent Al-doped ZnO (1–10 at.% Al) films were synthesized by solid-phase pyrolysis at 700 °C. Synthesized Al-doped ZnO films were investigated by X-ray diffraction (XRD), scanning and transmission electron microscopy (SEM, TEM). All obtained materials were crystallized into the wurtzite structure, which was confirmed by XRD. The material crystallinity decreases with the introduction of aluminum. SEM and TEM showed that the films are continuous and have a uniform distribution of nanoparticles with an average size of 15–20 nm. TEM confirmed the production of Al-doped ZnO films. The transmittance of Al-doped ZnO films in the range of 400–1000 nm is more than 94%. The introduction of 1% Al into ZnO leads to a narrowing of the band gap compared to ZnO to a minimum value of 3.26 eV and a sharp decrease in the response time to the radiation exposure with a wavelength of 400 nm. An increase in aluminum concentration leads to a slight increase in the band gap, which is associated with the Burstein–Moss effect. The minimum response time (8 s) was shown for film containing 10% Al, which is explained by the shortest average lifetime of charge carriers (4 s).

## 1. Introduction

Currently, the interest in transparent crystalline films of semiconductor oxides has increased, as they are used for optoelectronic and photovoltaic applications. Highly stable thin films of metal oxides with a transmittance of more than 80% in the visible region are one of the promising materials [1,2,3]. The most common materials with necessary properties are indium oxide and indium tin oxide (ITO). However, due to the high cost of these materials, the investigation for cheaper alternatives is relevant. Among the most affordable and inexpensive materials, zinc oxide can be noted. It is a direct-band semiconductor with n-type conductivity; its band gap is 3.37 eV [4]. ZnO-based films have high transparency and low resistance; they are widely used in optoelectronic devices such as solar cells [5], lasers and LEDs in the ultraviolet (UV) light range [6], and optical waveguides [7]. ZnO films are also used to create visible blind UV sensors [8,9], which are used for pollution monitoring, leak detection and other applications.

Previous studies have shown that the structural, optical, electrical, and photovoltaic properties of film materials based on zinc oxide are affected by various factors such as the method and conditions for the material formation, as well as the additives introduced [10,11]. The introduction of rare-earth elements into the structure of zinc oxide leads to the production of thin-film materials with low resistivity, which is promising for photovoltaic solar cells [12,13], but these materials are expensive. Doping with transition metals (Fe, Co, Ni, Mn, etc.) makes it possible to obtain nanostructured ZnO films applicable in optoelectronics and optical devices [14,15,16]. However, the use of additives of cobalt, manganese and iron ions leads to the formation of mixed oxides and deterioration of the material properties. Promising additives that increase the transparency and conductivity of ZnO films are elements of the 13th group of the periodic table of chemical elements, such as B, Al, Ga, In, [17]. Among the elements of the 13th group, Al is the most accessible, cheap and non-toxic additive. In addition, Al-doped ZnO film has better mechanical, thermal, and chemical stability [18]. Comparative characteristic of films based on zinc oxide obtained by various methods are presented in Table 1.

According to Table 1, Al-doped zinc oxide films have high transparency and the smallest crystallite size. Small concentrations of entered aluminum also contribute to the band gap narrowing. Co-doped composite materials have properties comparable with those of doped materials, and some characteristics are even worse (for example, the band gap is bigger), which allows the conclusion of the advantage of doping in this case. Due to the low cost, stability, good optical and electrical properties, Al-doped ZnO films can be an excellent replacement for ITO. Therefore, in this work, this additive was chosen for the formation of conductive transparent thin films.

Zinc oxide films can be synthesized by various methods, such as sol–gel [25,33], radiofrequency sputtering [27], atomic layer [28], chemical bath [29] deposition, hydrothermal [34], and others. Article [22] describes the synthesis of Al-doped zinc oxide films by the sol–gel method. It was established that the obtained materials are polycrystalline and high-intensity diffraction peaks correspond to the hexagonal structure of ZnO wurtzite. The electrical resistivity of Al-doped ZnO films is lower than that of pure zinc oxide. The minimum value of the electrical resistivity of the 1 at.% aluminum-doped ZnO film is 3.2 × 10^−2^ Ohm∙cm. It was shown that all films have a transmittance of more than 80% in the visible light range and the transmittance of Al-doped ZnO films is higher than that of undoped ones. The introduction of an additive (0.5, 1, 5%) leads to an increase in the band gap, which ranged from 3.34 to 3.62 eV for doped samples. Paper [23] presents the study of thin polycrystalline Al-doped zinc oxide films obtained by spray pyrolysis. All films have high transparency in the visible light range (380–850 nm); the transmittance is more than 90%. With the introduction of 5 at.%. Al^3+^, the optical band gap decreases to 3.27 eV compared to pure ZnO. The minimum resistance value is 6.246 × 10^−3^ Ohm∙cm for films containing a 2% additive.

One of the simple, inexpensive and promising films synthesis methods is solid-phase pyrolysis, which allows the obtention of films of various compositions: ZnO-SnO_2_ [35,36,37], Co_3_O_4_-ZnO [38]. By this method, using heat treatment at 600 °C, transparent ZnO films containing 1 and 3% Al^3+^ were obtained [39]; they had a transmittance of more than 90%. It was also found that with an increase in the calcination temperature from 600 to 700 °C, the band gap decreases from 3.32 to 3.28 eV, respectively [40]. Despite the studies conducted, a systematic study is still required showing the effect of Al doping concentration on the optical and photosensitive properties of ZnO.

In this article, we report on the morphological, phase, optical and photosensitive properties of thin Al-doped ZnO films with a content of 1–10 at.% Al obtained by solid-phase pyrolysis at 700 °C.

## 2. Materials and Methods

### 2.1. Preparation of ZnO and Al-Doped ZnO Thin Films

To obtain pure zinc oxide and Al-doped ZnO, synthesis from inorganic precursors was implemented; zinc acetate dihydrate Zn(CH_3_COO)_2_∙2H_2_O (analytical grade, A.G.), nonahydrate aluminum nitrate Al(NO_3_)_3_∙9H_2_O (A.G.), and abietic acid (A.G.) were used. Synthesis was carried out according to the previously described method [39]. At the first stage, an intermediate product was obtained in the melt—organic salts of aluminum and zinc. The resulting intermediate, which is a powder, was dissolved in an organic solvent. At the second stage, the solution of the intermediate product was applied by the dip-coating method to pre-prepared glass, quartz, polycore and silicon substrates with a size of 10 × 10 mm^2^ and dried. For this study, the solution was applied to the substrates three times to ensure the desired film thickness of about 100 nm. Each layer was dried for 10–15 min first in the air and second in the drying cabinet. Material heat treatment was carried out at 700 °C in the air for 2 h to remove the organic compounds. As a result of the synthesis, nanocrystalline thin film ZnO materials with a content of 1, 3, 5, 10 at.% Al^3+^ were obtained, which have the designations 1Al-ZnO, 3Al-ZnO, 5Al-ZnO and 10Al-ZnO. Undoped ZnO film was also synthesized for comparison.

### 2.2. Characterization

The obtained film materials were studied by thermogravimetry (TGA) and differential thermal analysis (DTA) using a synchronous thermal analysis device TG-DTA/DSC STA 449 °C/4 G Jupiter Jupted (Netzsch—Geratebau GmbH, Selb, Germany). Heating was carried out in air up to 800 °C at a speed of 10°/min.

The structural characterization of synthesized Al-doped zinc oxide films was carried out by X-ray diffraction (XRD) on an ARL’XTRA diffractometer (Thermo Scientific, Ecublens, Switzerland), monochromatic radiation CuKα_1_ (λ = 1.5406 Å). The particle size was calculated from the coherent scattering regions using the Scherrer equation [19]. Dislocation density (δ) and strain (ε) were calculated using standard formulas [41].

The morphology of the surface was studied using scanning electron microscopy (SEM) SEM NovaNanoLab 600 microscope (FEI, Eindhoven, The Netherlands) at 10 kV.

The shape and the size of crystallites and the elemental composition of thin films were studied by transmission electron microscopy (TEM), scanning transmission electron microscopy (STEM) and energy dispersive X-ray spectroscopy (EDX) using a Multi-purpose Electron Microscope JEM-F200 (JEOL, Akishima, Tokyo, Japan), operating at an accelerating voltage of 200 kV. The JEM-F200 is equipped with a cold field emission electron gun. For both TEM and EDX measurements, JEOL EM-01361RSTHB (JEOL, Akishima, Tokyo, Japan), double-tilt beryllium specimen holder was used.

TEM images were recorded at magnifications from 12,000× to 1,000,000× with a 500 to 2000 ms exposure on a high-resolution CMOS AMT camera.

EDX was performed with a Bruker Xflash 6T/60 Quantax 400-STEM system with 4000 channels, including an energy-dispersive Peltier-cooled XFlash detector, a 0.45 mm detector thickness and a −25 °C working temperature. EDX Mapping was performed with a 200 kV primary energy at 10 eV with a 16 to 32 s dwell time and a total measurement time from 6 to 15 min.

The optical properties of films on quartz substrates were studied using optical transmittance spectra obtained on the UV-1100 ECOVIEW spectrophotometer (Mapada Instruments Co, Shanghai, China) in the wavelength range of 200–1000 nm. The band gap energy was found according to absorption edge analysis [42].

The kinetics of the obtained films photoconductivity was studied using equipment [38,43] which allows the measurement of the photoresponse of the studied film by radiation from LED with a wavelength of 400 nm (GNL type, Ningbo, Zhejiang, China). The photoconductivity was controlled by a digital multimeter (Tektronix DMM4050, Keithley Instruments, Cleveland, OH, USA). The response time was determined at the level of 90% of the maximum change in photosensitivity (t0.9) under continuous exposure to LED radiation, and then the average lifetime of charge carriers was calculated (τ) [44].

## 3. Results and Discussion

### 3.1. DSC-TGA

**Figure 1:**
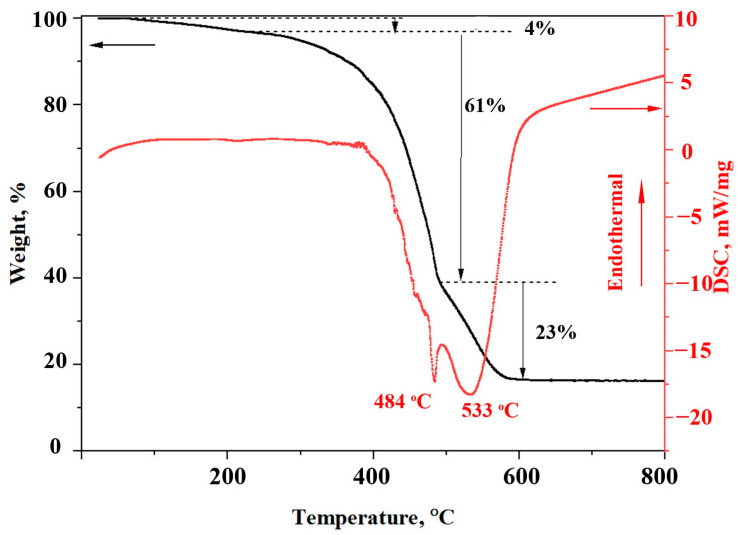
TGA and DSC of intermediate product 10Al-ZnO.

DSC-TGA of the obtained intermediate product 10Al-ZnO was carried out. The heating process of the sample took place in the temperature range from 25 to 800 °C. TGA and DSC curves are presented in Figure 1. On the TGA curve, it is possible to detect three stages associated with changes in the material mass. At the first stage of 25–245 °C, a mass loss of 3.6% occurs, which may be associated with the evaporation of adsorbed water. At the second temperature stage of 245–491 °C, the mass decreases by 60.5%. This can be explained by the combustion process of organic zinc and aluminum salts, during which a significant amount of energy is released, which corresponds to an exothermic peak at 484 °C on the DSC curve. At the last stage, in the temperature range of 491–600 °C, the mass loss is 23.6%. The exothermic peak in the region of 533 °C on the DSC curve is due to the formation of new bonds in the crystal lattice of the ZnO wurtzite phase. The total mass loss of the sample is 88%. In other studies, the gradual formation of Al-doped ZnO materials by chemical methods is also observed. Thus, when films are obtained by the precipitation method, the crystallization of ZnO occurs at temperatures over 400 °C, and the optimal synthesis temperature of the completely crystalline phase is 600 °C [45]. Based on the obtained DSC-TGA results, it can be concluded that the production of Al-ZnO films can be carried out at a temperature of 700 °C.

### 3.2. XRD

**Figure 2:**
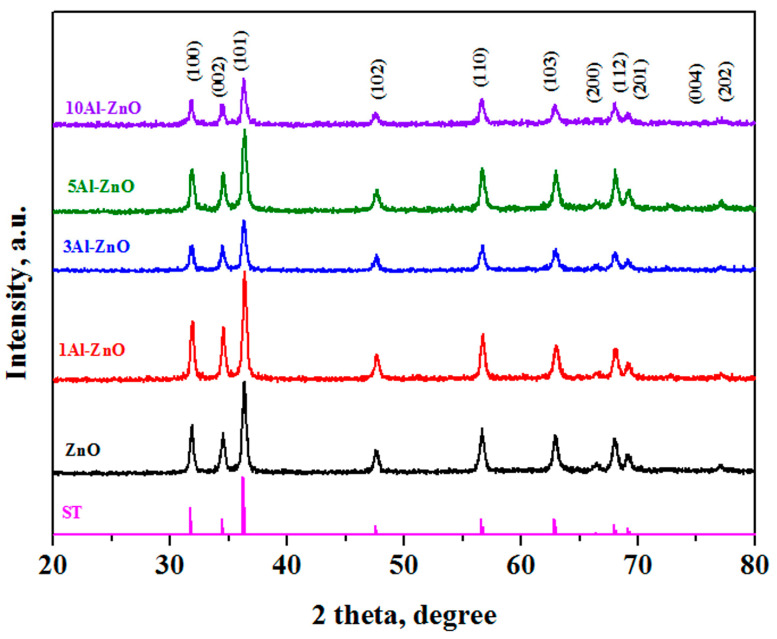
XRD patterns of synthesized materials and standard sample from the database (curve ST).

The synthesized materials were investigated by the XRD method (Figure 2). The presence of narrow, clear, well-distinguishable diffraction peaks (100), (002), (101), (102), (110), (103), (200), (112), (201), (004), (202) correlates with the hexagonal structure of wurtzite, as expected for zinc-oxide-based materials [29,33]. No impurity phases were found, which emphasizes the purity of the obtained materials. When Al^3+^ ions are added, there is a slight shift in the position of the peaks towards large Bragg angles compared to pure ZnO, which is clearly visible at the maximum peak (101). Deviations of the diffraction peak indicate that the aluminum ion was doped into the crystal lattice of the ZnO film.

Particle sizes, dislocation density (δ) and strain (ε) were calculated from the XRD data (Table 2). Since the ionic radius of aluminum (0.54 Å) is smaller than the ionic radius of zinc (0.74 Å [32]), the replacement of zinc ion with aluminum ion causes lattice compression and a decrease in lattice parameters. This trend is observed at low Al concentrations (up to 2%) [46]. After saturation of wurtzite, aluminum ions can be embedded in the interstices of the lattice, which can lead to the formation of a large number of defects. At the same time, amorphization occurs, which explains the decrease in the peak intensity in the XRD pattern. There is also a decrease in the crystallite size compared to pure zinc oxide.

The dislocation density is caused by the defect and distortion occurrence in the crystal lattice. The appearance of strain is associated with the formation of dislocations at the grain boundary [47]. As can be seen from the results, with an increase in the aluminum concentration in the materials, the dislocation density and stress increase, which confirms the increase in defects. The obtained results are consistent with the previously presented data [48].

As one can see from the presented data, films obtained by solid-phase pyrolysis do not have a preferred orientation of crystallite growth. When aluminum is added, a decrease in crystallinity is observed. It is interesting that the film characteristics depend on the synthesis method. For example, the undoped ZnO film has a preferred (002) orientation when synthesized by spray pyrolysis [26]. Al doping causes a loss of preferential orientation of the films and a decrease in the crystalline quality of the films. A similar pattern is observed for Al-doped ZnO films deposited at room temperature by a cost-effective sol–gel dip-coating technique [49], RF sputtering [27], dip coating process processed under thermal shock conditions [33], and chemical spray pyrolysis [50].

### 3.3. SEM

**Figure 3:**
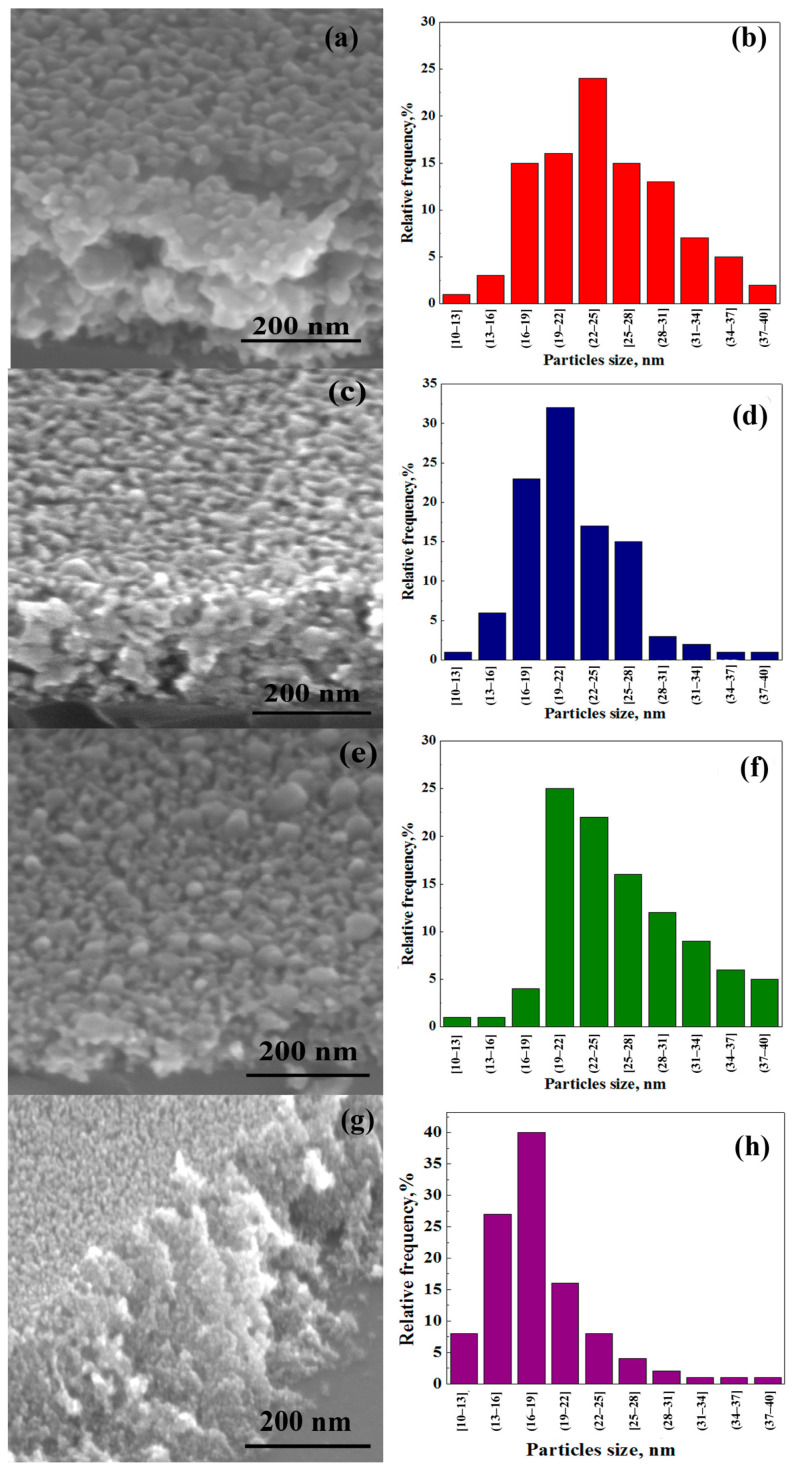
SEM photos of surface (**a**,**c**,**e**,**g**) and distribution of nanocrystallites (**b**,**d**,**f**,**h**) in 1Al-ZnO (**a**,**b**), 3Al-ZnO (**c**,**d**), 5Al-ZnO (**e**,**f**), 10Al-ZnO (**g**,**h**) films.

Since the microstructure affects the optical properties, it is important to investigate the morphology of the film surface. SEM microphotographs of Al-ZnO films are shown in Figure 3. The film surface is solid and has a uniform distribution of well-formed crystallites. The cross-sectional SEM photo shows that the film thickness is 70–210 nm. The SEM photo is assigned for three-layer films; the absence of boundaries between layers is shown, which allows the conclusion that the selected synthesis method is advantageous for obtaining solid coatings. The average particle size is in the range of 16–25 nm depending on the aluminum content in the obtained films—Figure 3b,d,f,h.

A slightly different surface structure is observed for films synthesized by other methods. Al-doped ZnO films processed under thermal shock conditions [33] have a wrinkled micro-rod structure. The surface of Al-doped ZnO films prepared using sol–gel consist of nanostructured particles and clusters created from a combination of these particles [51]. Films surface obtained by chemical spray pyrolysis consist of agglomerates formed by polycrystals [50]. The film surface similar to this work according to SEM morphological images was obtained for Al-doped ZnO films grown by high-speed atmospheric atomic layer deposition. It is interesting that the sample has elongated grains parallel to the substrate; the crystallite size is around 20–27 nm [28].

### 3.4. TEM

**Figure 4:**
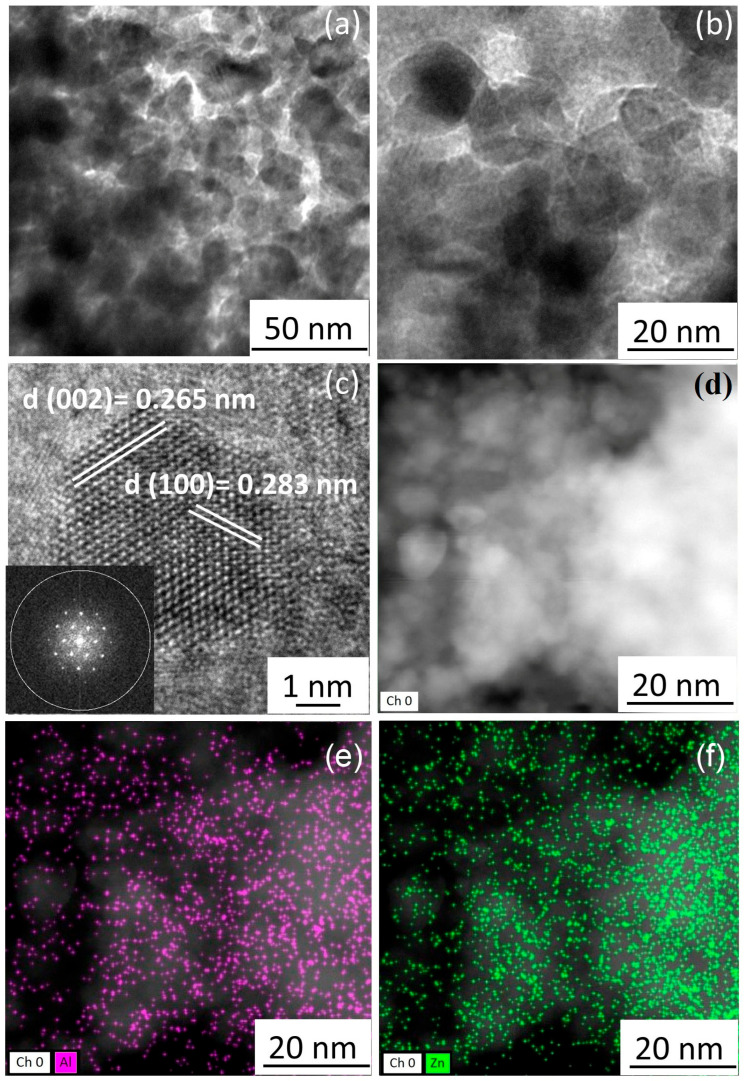
TEM investigation of Al-ZnO film: TEM photos with different magnification (**a**,**b**), HRTEM with FFT picture inset (**c**), STEM image (**d**) with element composition and Al and Zn mapping analysis (**e**,**f**).

TEM photos of the 10Al-ZnO film with different magnification are shown in Figure 4. It was shown that the synthesized films consist of crystallites with a size range of 15–20 nm, which is consistent with the data obtained by calculation for coherent scattering regions and from statistical processing of SEM photographs.

Based on Figure 4c, the elemental composition of the obtained film materials was determined by the EDX (Figure 4d–f) method. According to the elemental analysis, possible elements were found in the material: Al—5 wt.%, Zn—82 wt.%, O—13 wt.%. Thus, when converted to metal fractions of the content, Al:Zn = 12:88, which confirms the amounts of metals used in the synthesis, taking into account the EDX error (3%). According to Figure 4d,e, it can be concluded that aluminum atoms are distributed in zinc oxide, which excludes the presence of a composite mixture of two oxides.

Figure 4c shows the interplane distances characteristic of ZnO: d(002) 0.264 nm and d(100) 0.283 nm. The interplane distances characteristic of Al_2_O_3_ were not found in the photo, which allows the conclusion that a material consisting of zinc oxide with the addition of aluminum, rather than a mixture of two oxides, was obtained.

### 3.5. Optical Properties

**Figure 5:**
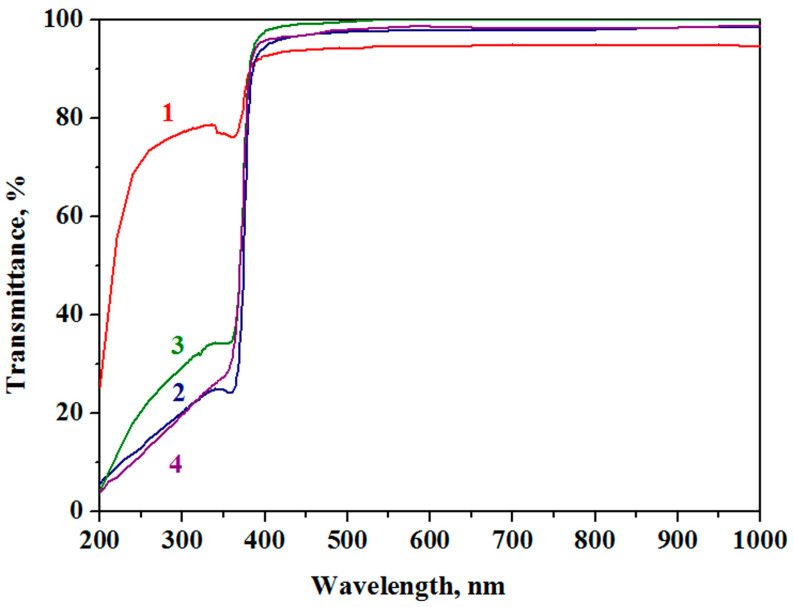
Optical transmittance spectra 1Al-ZnO (Curve 1), 3Al-ZnO (Curve 2), 5Al-ZnO (Curve 3) and 10Al-ZnO (Curve 4).

Optical transmittance spectra in the range of 190–1000 nm for Al-doped ZnO materials are shown in Figure 5. It is established that the obtained films are optically transparent in the range from 400 to 1000 nm with a transmittance of more than 94% for all materials; 1Al-ZnO has the highest transmittance in the UV light range. However, in the visible light range, the transmittance of this material is less prominent compared to that of the samples containing a larger amount of the additive. The highest transmittance in the visible light range of 98% is characteristic of the 5Al-ZnO film.

**Figure 6:**
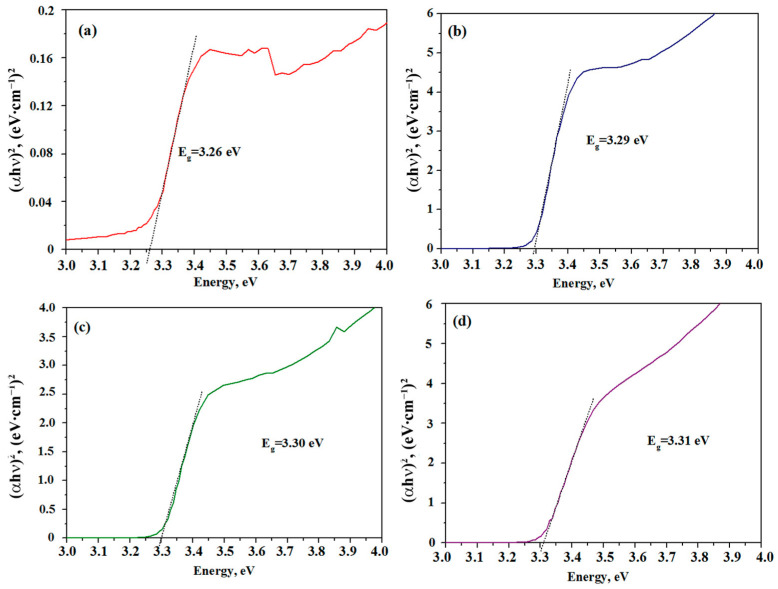
Tauc plots for 1Al-ZnO (**a**), 3Al-ZnO (**b**), 5Al-ZnO (**c**) and 10Al-ZnO (**d**) thin films.

The band gap (Eg) is determined from the dependence (αhν)^2^ of the photon energy, as shown in Figure 6. By extrapolating the direct part of the (αhv)^2^ to the point α = 0, the required value is found. The band gap is 3.26 (a), 3.29 (b), 3.30 (c) and 3.31 (e) eV for Al-doped ZnO films containing 1, 3, 5 and 10 at.% Al, respectively. All values are slightly smaller than that of the band gap of pure zinc oxide—3.37 eV. The authors of other publications [19,20,21,22,23] also showed a decrease in the band gap for Al-doped materials compared with the undoped ZnO films. An increase in the Al^3+^ concentration can lead to both a decrease [19,21] and an increase [24,52,53] in the band gap. For Al-doped ZnO materials synthesized in this work, an increase in the band gap with an increase in the aluminum concentration can be explained by the Burstein–Moss effect. Also, an increase in the aluminum concentration (aluminum has a higher charge than zinc, which allows its usage as an electron donor) leads to its inclusion in the crystal structure of zinc oxide, causing an increase in the transport path of charge carriers.

### 3.6. Photosensitive Properties

**Figure 7:**
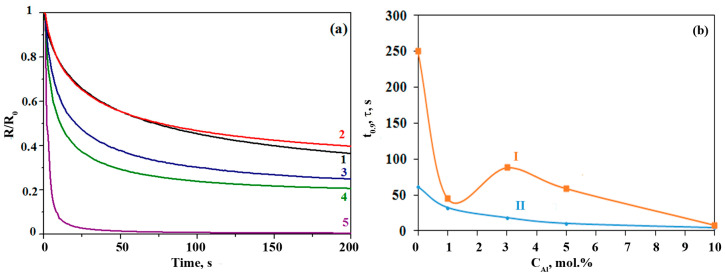
Time dependence of (**a**) normalized photosensitivity and (**b**) response time (I) and average relaxation time of charge carriers (II) for films ZnO (1), 1Al-ZnO (2), 3Al-ZnO (3), 5Al-ZnO (4), 10Al-ZnO (5).

Photosensitive properties of all obtained Al-ZnO films were studied. The photoconductivity parameters were studied under the influence of LED radiation with a wavelength of 400 nm. Figure 7 shows the time dependences of the normalized photosensitivity, response time (t_0.9_) and the average relaxation time of charge carriers (τ) for Al-ZnO films.

Studies have shown that pure zinc oxide has a long response time t_0.9_ equal to 250 s. But with the addition of a 1% aluminum, the response time drops sharply to 45 s. This may be due to a decrease in the band gap in this material compared to the band gap of ZnO. Further, with an increase in the concentration of aluminum to 3%, the response time increases by almost twofold (88 s). With a further increase in the aluminum content to 5% and 10%, a monotonous decrease in response time to 8 s occurs. The latter is explained by an exponential decrease in the average lifetime of charge carriers from 61 s for ZnO films and up to 4 s for 10Al-ZnO films.

This behavior of the photoconductivity parameters is due to the fact that aluminum is added to ZnO; crystallites with different characteristics are formed, which is well reflected in the appearance of the Urbach tail on the optical absorption curves in Figure 6a. In addition, the kinetics of the film photoresponse with an increase in the concentration of aluminum in ZnO begins to change, which indicates the emergence of another mechanisms of generation–recombination of charge carriers in ZnO films with the addition of Al. The latter may be due to the occurrence of a strong surface electric field at the interface [35,37,38,54]. Doping zinc oxide with aluminum leads to a decrease in the work function of the electron from doped Al-ZnO nanocrystals. In study [55], it was found that the work functions of ZnO and Al-doped ZnO are 5.076 eV and 4.978 eV, respectively. When Al-ZnO crystallites come into contact with undoped ZnO crystallites due to the difference in the work functions, electrons transfer from Al-ZnO crystallites to ZnO crystallites, and a surface potential is formed at their boundary. In general, an increase in the concentration of aluminum in ZnO films leads to an exponential decrease in the average relaxation time of charge carriers. However, the response time has a minimum for 1Al-ZnO films, which may be due to both a decrease in the band gap and higher values of surface electric fields in films with a small additive concentration [37].

The obtained results make it possible to use the obtained materials in UV radiation monitoring sensors, in solar cells, both as a UV-absorbing layer and for use as an antireflection layer in order to increase the efficiency of solar radiation conversion.

## 4. Conclusions

Al-doped ZnO films with different concentrations of aluminum from 1 to 10 at.% were obtained by solid-phase pyrolysis. The resulting films are nanoscale and homogeneous, crystallized into the wurtzite structure. The films are formed by nanocrystallites with a size of 15–20 nm. It was shown that with an increase in the aluminum concentration up to 10%, a decrease in particle sizes was observed. All films were transparent in the visible light range, with the highest transmittance of 98% for the 5Al-ZnO film.

The study of photosensitive properties showed that for films with the highest Al content (10%), there was a decrease in the photoresponse time to 8 s, and the average lifetime of charge carriers is 4 s, which is explained by the emergence of new mechanisms of generation–recombination of charge carriers in ZnO films with the addition of Al. At the same time, when a 1% aluminum was added to ZnO, the response time dropped to 45 s compared to the response time of both pure ZnO (250 s) and 3Al-ZnO (88 c). This is due to a sharp decrease in the band gap of the 1Al-ZnO film and the presence of a strong surface electric field.

## Figures and Tables

**Table 1:** Comparative characteristics of the properties of films based on zinc oxide obtained by various methods.

Material	Synthesis Method	Particle Size/Film Thickness, nm	Optical Properties		Ref.
			Transmittance, %	Band Gap, eV	
0.5 at.%Al-ZnO	Sol–gel and further implantation of Al	22.24/350	82	3.393	[18]
1 mol.%Al-ZnO	Sol–gel	20/-	80	3.31	[19]
3 mol.%Al-ZnO		20/-	80	3.315	
5 mol.%Al-ZnO		20/-	80	3.305	
2 wt.%Al-ZnO	RF magnetron sputtering	27/-	95	3.36	[20]
2.1 at.%Al-ZnO	Excimer laser annealing	-/180	92	3.64	[21]
0.5 at.%Al-ZnO	Sol–gel	38/-	90	3.58	[22]
1 at.%Al-ZnO		-/-	83	3.41	
5 at.%Al-ZnO		64/-	87	3.62	
1 at.%Al-ZnO	Spray pyrolysis	-/-	90	3.3	[23]
2 at.%Al-ZnO			98	3.25	
3 at.%Al-ZnO			98	3.28	
4 at.%Al-ZnO			90	3.27	
5 at.%Al-ZnO			90	3.29	
Mg_0.03_Al_0.03_ZnO_0.94_	RF magnetron sputtering	-/550–750	87.2	3.7	[24]
Mg_0.03_Ga_0.03_ZnO_0.94_			89.2	3.87	
Mg_0.03_In_0.03_ZnO_0.94_			79.2	3.43	
0.5 at.%Al-ZnO	Sol–gel	-/-	90	3.25	[25]
1 at.%Al-ZnO	Chemical spray pyrolysis	-/-	80	3.55	[26]
2 at.%Al-ZnO			78		
4 at.%Al-ZnO			70		
10 at.%Al-ZnO	Radiofrequency sputtering	35.6/230	80	4.30	[27]
Al-ZnO	Atomic layer deposition	20.5/60	80	3.265	[28]
Al/ZnO nanorods	Chemical bath deposition	48/256	70	3.25	[29]
1 mol.%Al-ZnO	Sol–gel	-/150	85	3.28	[30]
5 at.%Al-ZnO	Sol–gel	12.28/525	95	3.33	[31]
2 at.%Al-ZnO	Sol–gel	-/326	80	3.38	[32]
1 at.%Al-ZnO	Sol–gel	10.4/296.59	90	3.261	[33]

**Table 2:** Measurements of crystallite sizes and lattice parameters.

Sample Name	Crystallite Size, nm	δ × 10^3^ (nm^−2^)	ε × 10^3^
ZnO	18	2.67	5.615
1Al-ZnO	17	3.34	6.513
3Al-ZnO	17	3.45	6.805
5Al-ZnO	16	3.80	7.082
10Al-ZnO	13	5.47	8.443

## Data Availability

Not applicable.
